# High Blood Pressure Effects on the Blood to Cerebrospinal Fluid Barrier and Cerebrospinal Fluid Protein Composition: A Two-Dimensional Electrophoresis Study in Spontaneously Hypertensive Rats

**DOI:** 10.1155/2013/164653

**Published:** 2013-01-21

**Authors:** Ibrahim González-Marrero, Leandro Castañeyra-Ruiz, Juan M. González-Toledo, Agustín Castañeyra-Ruiz, Hector de Paz-Carmona, Rafael Castro, Juan R. Hernandez-Fernaud, Agustín Castañeyra-Perdomo, Emilia M. Carmona-Calero

**Affiliations:** ^1^Departamento de Anatomía, Facultad de Medicina, Universidad de La Laguna, Ofra s/n, 38071 La Laguna, Tenerife, Spain; ^2^Departamento de Farmacología, Facultad de Medicina, Universidad de La Laguna, Ofra s/n, 38071 La Laguna, Tenerife, Spain; ^3^Instituto de Investigación y Ciencias de Puerto del Rosario, c/Tenerife n 35, 35600 Puerto del Rosario, Fuerteventura, Spain; ^4^Departamento de Fisiología, Facultad de Medicina, Universidad de La Laguna, Ofra s/n, 38071 La Laguna, Tenerife, Spain

## Abstract

The aim of the present work is to analyze the cerebrospinal fluid proteomic profile, trying to find possible biomarkers of the effects of hypertension of the blood to CSF barrier disruption in the brain and their participation in the cholesterol and **β**-amyloid metabolism and inflammatory processes. Cerebrospinal fluid (CSF) is a system linked to the brain and its composition can be altered not only by encephalic disorder, but also by systemic diseases such as arterial hypertension, which produces alterations in the choroid plexus and cerebrospinal fluid protein composition. 2D gel electrophoresis in cerebrospinal fluid extracted from the cistern magna before sacrifice of hypertensive and control rats was performed. The results showed different proteomic profiles between SHR and WKY, that **α**-1-antitrypsin, apolipoprotein A1, albumin, immunoglobulin G, vitamin D binding protein, haptoglobin and **α**-1-macroglobulin were found to be up-regulated in SHR, and apolipoprotein E, transthyretin, **α**-2-HS-glycoprotein, transferrin, **α**-1**β**-glycoprotein, kininogen and carbonic anhidrase II were down-regulated in SHR. The conclusion made here is that hypertension in SHR produces important variations in cerebrospinal fluid proteins that could be due to a choroid plexus dysfunction and this fact supports the close connection between hypertension and blood to cerebrospinal fluid barrier disruption.

## 1. Introduction

Cerebrospinal fluid is a functional system closely connected to the brain, and changes or variations in the CSF may mean an alteration in the brain expressed by encephalic disorders. However, the composition of CSF may also be altered by systemic diseases, such as arterial hypertension, and cerebral ventricular dilatation, changes in CSF protein, and variations of the choroid plexus and other circumventricular organs (CVO) have been described in spontaneously hypertensive rats (SHR) [1–5]. Therefore, SHR develop hydrocephalus and experimental studies explain that hydrocephalus induces alterations in CSF since there are disturbances, in the hydrocephalic brain, of oxidative metabolism and neurotransmission and perhaps damage to periventricular cells, particularly when intracranial pressure is elevated [6]. The sharp increase in systemic blood pressure only causes an acute increase in CSF pressure in normotensive animals and not in hypertensive patients [6]. The CSF pressure of SHR showed greater protection to the acute effects of phenylephrine than in control Wistar-Kyoto (WKY) rats, but a permeability increase of the blood to cerebrospinal fluid barrier to sucrose in rats with chronic hypertension was detected [7]. The blood-brain barrier (BBB) was found to be resistant to the passage of sucrose and lanthanum in both SHR and WKY rats, indicating that BBB integrity was maintained, however, there is a reduced brain uptake of sucrose in SHR compared to WKY, which is consistent with decreased brain capillary density and reduction of cerebral blood flow (CBF) in SHR compared to WKY [7]. Furthermore, in a previous study, increases of transthyretin (TTR) monomer and S-100 *β* in blood have been described in SHR which means a disruption of the brain barriers [8]. 

The brain is one of the first target organs of high blood pressure, which is the main modifiable risk factor for stroke. Hypertension causes a progressive increase in cerebral blood flow in the blood vessels of the brain that perform complex and dynamic relationships between blood pressure and brain function. High blood pressure can accelerate atherosclerotic changes and affect cerebral autoregulation [9]. On the other hand, epidemiological studies link cardiovascular risk factors such as hypertension and high plasma cholesterol to dementia. The modulation of the degradation of amyloid precursor protein by the administration of cholesterol in cell cultures and animal models of beta-amyloid overproduction has been described [10] and a connection between inflammation, hypertension and beta-amyloid accumulation has also been reported [11]. 

Subsequently, as mentioned above, a previous work has reported that high blood pressure produces alterations in the circumventricular organs (CVO), ventricular dilation, variations in the protein composition of the CSF, an increase in the inflammatory process in the brain, and alterations of beta-amyloid metabolism. The aim of the present work is to analyze the protein composition of the CSF of the SHR and its relationship with the integrity of the blood to CSF barrier.

## 2. Materials and Methods

### 2.1. Animals

Thirty-six-month-old rats divided into two groups were used: a control group of 15 Wistar Kyoto (WKY) and a hypertensive group of 15 spontaneously hypertensive rats (SHR) from Charles River Laboratories España S.A. (Barcelona, Spain). The animals were maintained at a constant temperature of 21 ± 2°C and 55 ± 8% relative humidity on a normal 12–12-hour light-dark cycle. The systolic blood pressure (SBP) of SHR and WKY rats at 6 months of age was measured indirectly using the tail-cuff method in conscious rats.

### 2.2. Cerebrospinal Fluid Samples

Animals were anesthetized with chloral hydrate (400 mg/kg) and placed in a stereotaxic frame. CSF was extracted from the cistern magna of the SHR and WKY rats. The CSF was centrifuged (4,000 g for 4 min) to remove blood contamination from the puncture point. Cleared CSF was stored at −80°C. 

### 2.3. Immunohistochemistry

The brains of four rats from each group were fixed with Bouin fixative, embedded in paraffin, and cut in four (A, B, C, and D) parallel series of section at a thickness of 10 *μ*m. The brain areas containing the choroid plexus (CP) of the A series were stained with hematoxylin-eosin (H-E). The sections of the other series (B, C, and D) containing the CP, after deparaffinization and rehydration, tissues were treated with hot (85°C) 10 mM citrate buffer, pH 6, for 20 min. Sections were washed with distilled water and quenched with 3% hydrogen peroxide for 10 min at room temperature to eliminate endogenous peroxidase activity. After washing in 0.05 M Tris-buffered saline (TBS) pH 7.6, the sections were incubated with anti-rat IgG 1 : 200 (Sigma) for two hours, then the sections were washed in TBS and stained using 3.3-diaminobenzidine (DAKO). 

The immunohistochemical slides were converted into digital images by using a LEICA DMRB photomicroscope with a LEICA DC 300 F camera (Germany). Image analysis was completed in Image J (v. 1.43 u, NIH, Bethesda, MD, USA). The “Mean Gray Value” was measured from the selected areas for all stained tissues. This value gives the average stain intensity in grayscale units for all threshold pixels. The immunohistochemistry statistical study was conducted using the IBM SPSS statistic 19 software (one-way ANOVA).

### 2.4. *2D* Gel Electrophoresis in CSF

CSFs from SHR and WKY rats were pooled and samples were solubilized in buffer with 8 M urea, 4% CHAPS, 40 mM Tris, 65 mM DTE, 0.05% SDS, and 2% ampholytes. Isoelectrofocusing was performed using glass capillary tubes (1.5 mm id and 12 cm length) and capillary tubes were filled with solution containing 3% acrylamide, 7 M urea, 0.6% Triton X-100, 0.75% ampholytes pH 5–8, 0.22% ampholytes pH 3–10, and 0.22% ampholytes pH 7–9, 0.045% TEMED, and 0.08% APS were used to separate proteins in the 4–8 pH range. Samples containing approximately 100 *μ*g total proteins were applied to the base end of the tube gel and were resolved in cathode and anode buffers of 20 mM NaOH and 8.7 mM H_3_PO_4_, respectively. IEF was carried out in steps of 1 hr at 100 and then 300 V, followed by 17.5 hr at 1000 V and 30 minutes at 2000 V. The capillaries were then equilibrated for 15 minutes in reducing buffer containing 50 mM Tris.HCl [pH 8.8], 30% glycerol, 6 M urea, 2% SDS, and 1% DTT, followed by a blocking step for another 15 minutes in a similar buffer containing 2.5% iodoacetamide instead of DTT. The capillary gels were then transferred to the top of an 18 × 18 cm, 1.5-mm-thick, 10% polyacrylamide gel (SDS-PAGE) and embedded in 0.5% low-melting agarose containing a trace of bromophenol blue. SDS-PAGE was run at 15°C, initially at 20 mA for 15 min and then at 50 mA per gel until the blue front reached the bottom. Molecular mass markers (Sigma) were loaded onto the second dimension for external calibration. The protein spots were visualized in preparative gels by staining with the colloidal Coomassie stain. Gels were scanned using a UMAX scanner (Amersham Biosciences) and the images were analyzed with Melanie version 5.0 software (GeneBio, Geneva, Switzerland), which included spot detection, quantification, normalization, and data analysis. Matching corresponding spots across different gels was performed by comparative analysis of protein spots, and each of the matched protein spots was checked manually. The intensity volumes of the individual spots were normalized to the total intensity volume of all the spots present in each gel and were subjected to Kolmogorov statistical analysis to compare the normalized intensity volumes of the individual spots from the controls to those of the hypertensive group. Only differentially expressed proteins were excised and subsequently identified by a mass spectrometer (MS).

### 2.5. MALDI-TOF-MS

Protein spots were manually excised from stained gels and the tryptic in-gel digestion and desalting steps were performed using 96-well ZipPlates (Millipore, Bedford, MA, USA) according to the manufacturer's instructions. The resulting peptides were mixed with 1 *μ*L *α*-cyano-4-hydroxycinnamic acid (CHCA; 1 mg/mL) and spotted onto Anchorchip plates as described by the manufacturer (Bruker-Daltonics, Bremen, Germany). Peptide mass fingerprint spectra were measured on an Autoflex MALDI-TOF (Bruker-Daltonics) in a positive ion reflection mode and spectra in the 900-3, 200 Da range were recorded. The PMF data were submitted to the MASCOT search engine for protein identification using the Mascot database. The search parameters were set according to the following criteria: rattus for taxonomy, carbamidomethyl (C) for fixed modifications, oxidation (M) for variable modifications, and ±100 ppm for peptide ion mass tolerance.

## 3. Results

### 3.1. Hematoxylin-Eosin

**Figure 1:**
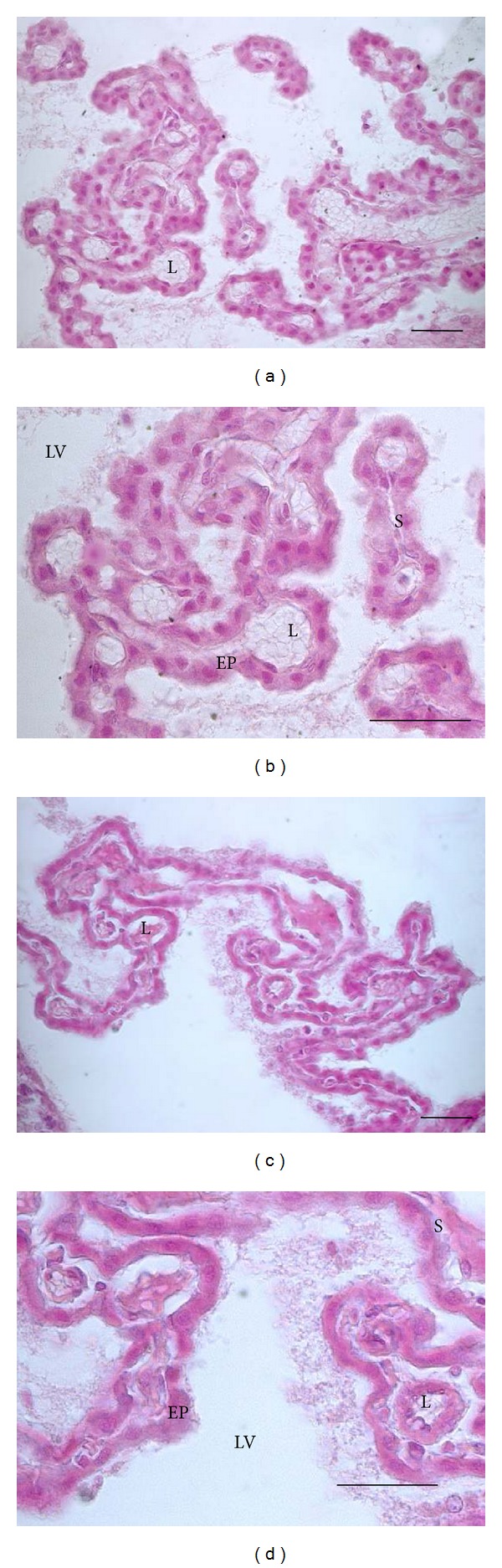
Coronal sections of rats showing choroid plexus of the lateral ventricle stained with hematoxylin-eosin: WKY rats, ((a) and (b)) and SHR ((c) and (d)). Abbreviations: L: lumen, EP: epithelium of the choroid plexus, S: stroma, and LV: lateral ventricle (bars = 30 *μ*m).

The WKY rats (Figure 1) show the typical structure of the choroid plexus, that is, the cubical morphology of the epithelial choroid plexus cells, little stroma, and small blood vessels. SHR show that the flattening of the choroid plexus epithelial cells and the stroma is greater in size, where the distance between the epithelium and choroidal vessels increased. The blood vessel diameter is also greater in SHR when compared to the controls. The morphology of CP becomes irregular, since continuity is lost in some areas between epithelial cells. Furthermore, the morphology of the nucleus has changed from circular to ellipsoid (Figure 1).

### 3.2. Immunohistochemistry

**Figure 2:**
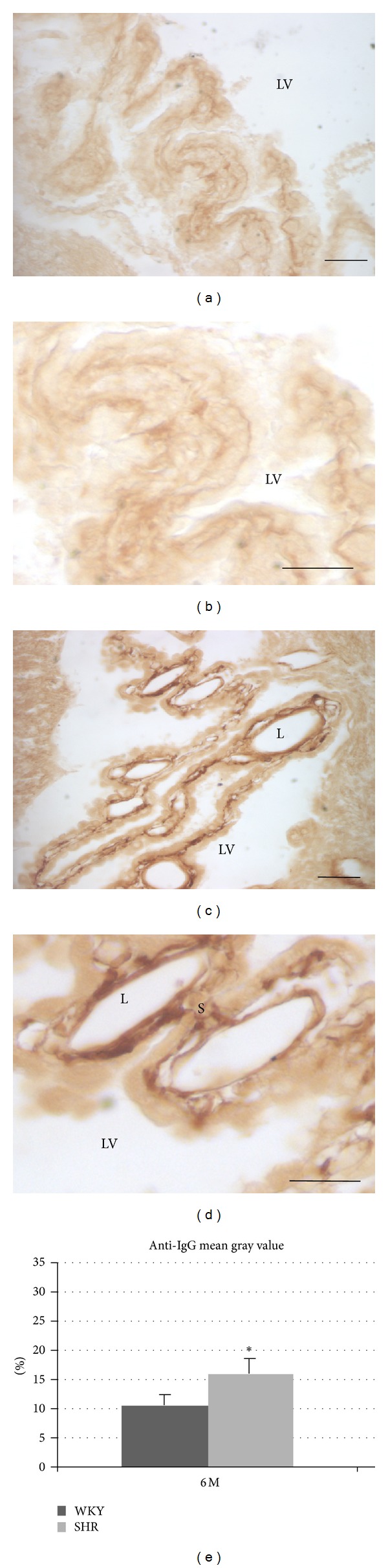
Coronal sections of rats showing choroid plexus the lateral ventricle showing of IgG immunostaining. (a) and (b): WKY rats, (c) and (d): SHR rats, (e): histogram showing the immunohistochemical quantification (mean gray value) for IgG in the choroid plexus of the lateral ventricle.Abbreviations: L: lumen, EP: epithelium of the choroid plexus, S: stroma, and LV: lateral ventricle. (escale bar: 30 *μ*m).

The IgG labeling in WKY rats was very low or almost undetectable in most of the choroid plexus tissues, and only a little amount of IgG located around blood vessels can be observed (Figure 2). By contrast, the IgG immunoreactive was clearly observed in the CP of SHR; the IgG was more intensive and distributed in a large number of cerebral vessels, in the stroma and basolateral membrane of the CP (Figure 2).

### 3.3. *2D* Eelectrophoresis

**Figure 3:**
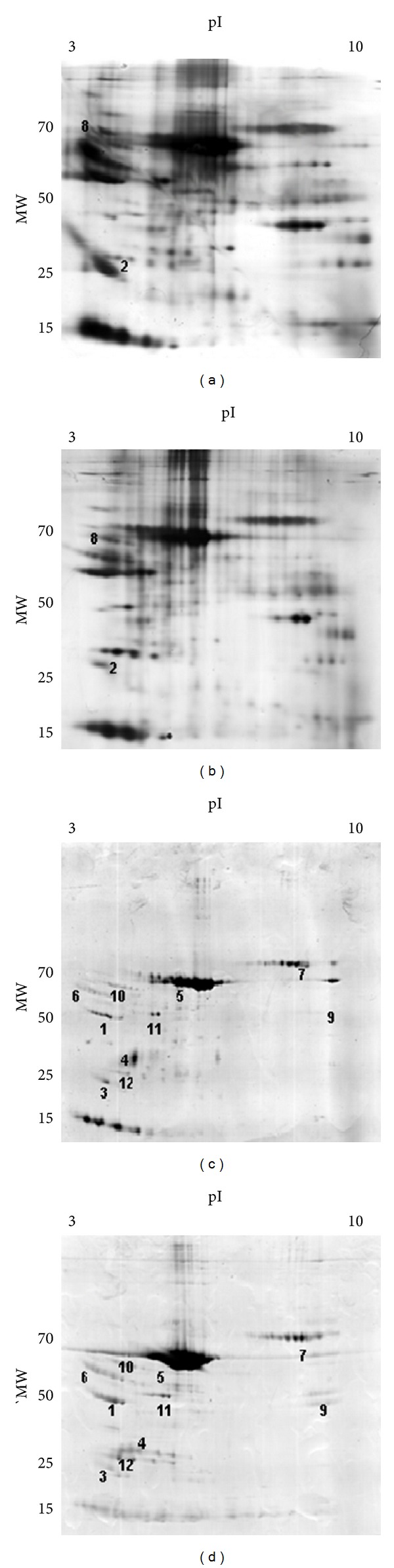
Two-dimensional gel electrophoresis using CSF from: (a) and (c): 6 month-old Wistar-Kioto rats; (b) and (d) 6 month-old SHR rats. (a) and (b) gels were obtained with silver staining and (c) and (d) were stained with Coomassie blue. The approximate isoelectric point (pI) and molecular weights (MW) are shown. The numbers in the gels correspond to the numbering in Table 1.

**Figure 4:**
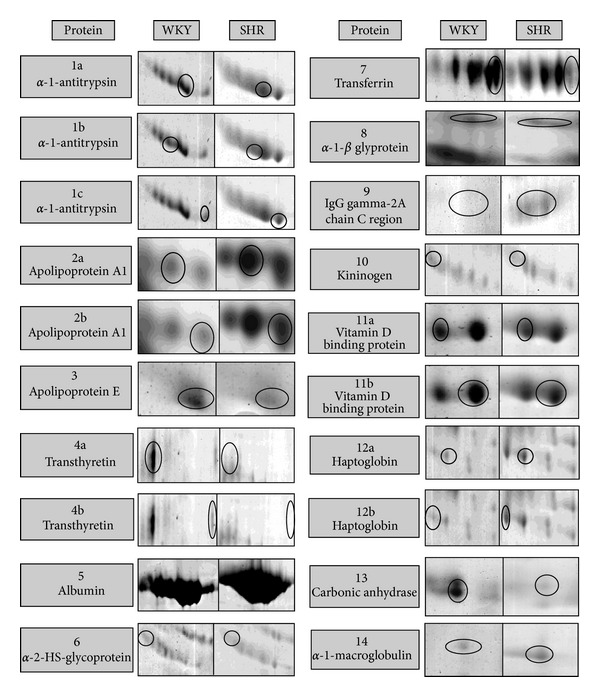
Zoom in two-dimensional gel of differential proteins.

**Figure 5:**
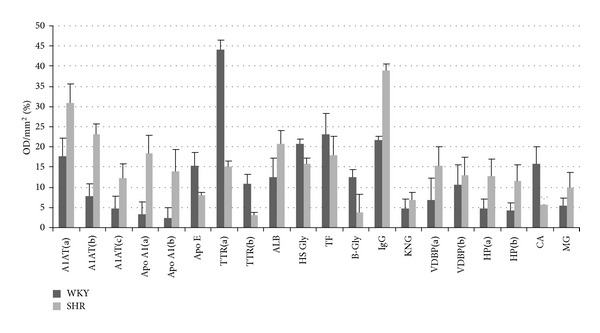
Differences in optic density between differential proteins of CSF from WKY and SHR. Abbreviations: A1AT: *α*-1-antitrypsin, Apo A1: apolipoprotein A1, Apo E: apolipoprotein E, TTR: transthyretin, ALB: albumin, HS Gly: *α*-2-HS-glycoprotein, TF: transferrin, B-Gly: *α*-1-*β* glyprotein, IgG: IgG gamma-2A chain C region, KNG: kininogen, VDBP: vitamin D binding protein, HP: haptoglobin, CA: carbonic anhydrase, and MG: *α*-1 macroglobulin.

The protein changes found were classified in three groups: group 1 correlated with the blood to CSF barrier (BCSFB), group 2 correlated with *β*-amyloid and cholesterol metabolism, and group 3 correlated with acute phase inflammatory process. The results showed different proteomic profiles between SHR and WKY; *α*-1-antitrypsin, apolipoprotein A1, albumin, immunoglobulin G, vitamin D binding protein, haptoglobin, and *α*-1-macroglobulin were upregulated in SHR and apolipoprotein E, transthyretin, *α*-2-HS-glycoprotein, transferrin, *α*-1*β*-glycoprotein, kininogen, and carbonic anhidrase II were downregulated in SHR (Figures 3, 4 and 5 and Table 1).

## 4. Discussion

The 2*D* gel electrophoresis of the CSF showed many protein variations in the CSF of SHR rats which are expressed by a different proteomic profile when comparing control and hypertensive rats, where some proteins are differentially expressed in the CSF of SHR compared to WKY. Six-month-old rats were chosen because, according to Zicha and Kuneš [13], there are the major differences between the blood pressure of SHR and WKY at this age. Among these variations, the most noteworthy changes found in the three groups are as follows.

### 4.1. Blood-to-CSF Barrier Related Proteins

Some authors have reported that chronic hypertension in SHR may cause more pronounced defects in the integrity of the blood-to-CSF barrier than the blood-brain barrier (BBB) [7, 14]. Transthyretin is found in CP and is secreted in the CSF [7, 14]. Low levels of TTR in SHR compared to WKY were found in this work; these differences in the blood-to-CSF barrier may explain the decrease of TTR in the CSF of SHR, allowing its passage from the CSF to the vascular space [7, 8, 14]. Therefore, this protein can be used to analyze the integrity of the BCSFB. Alternatively, the carbonic-anhydrase II (CA II) is abundantly expressed in the brain; it has been found by others in the CP and leaking into the CSF [15, 16]. The CA II level during CP development may be involved in early CSF secretion and the movement of water into the cerebral ventricles [17], secretion, and movement of water that appear to be altered in SHR since CA II is clearly lower in the results presented here. IgG immunoreactive was clearly greater in all of the CP structural components of the SHR;furthermore, IgG, albumin and haptoglobin are also stronger in the CSF of the SHR, which may be caused by damage to the BSLCR allowing their passage into the CSF [17–19]. In the proteomic study of the CSF of the SHR and WKY, albumin, IgG, and the two detected isoforms of haptoglobin were higher in SHR with respect to WKY rats (Table 1). These data support the idea that the hypertension produces an increase in some proteins derived from blood plasma due to a disruption of the BSLCR.

### 4.2. *β*-Amyloid and Cholesterol Metabolism

Apolipoprotein E (Apo E) is a molecule of the apolipoprotein family which is the main component of quilomicrones. Apo A1 is the most abundant apolipoprotein in plasma, almost all of which is present in HDL (cholesterol) and is about 90% and 65% of the protein fraction in HDL2 and HDL3 (cholesterol), respectively. Apo E and Apo A1 together with the TTR and vitamin D binding proteins are clearly implicated in the metabolism of *β*-amyloid and cholesterol [20]. *α*-1-macroglobulins are a family of glycoproteins that inhibit all four types of proteinases by a trapping mechanism [21]. Furthermore, the reaction of lecithin cholesterol acyltransferase (LCAT) with high density lipoprotein (HDL) is very important in reverse cholesterol transport [22, 23]. A decrease of Apo E in CSF of the SHR compared with WKY was found in the results here, but the proteins binding vitamin D, Apo A1, and *α*-1-macroglobulin are higher in the hypertensive group, which could explain the relationship between hypertension, inflammation, and amyloid pathology [11, 24]. 

### 4.3. Acute Phase Inflammatory Process

The CP is the main source of transferrin in the brain where it binds to the iron coming from the bloodstream [25]. Neuron survival requires iron, which is predominantly delivered by transferrin. The concentration of transferrin in CSF reflects brain iron availability and can serve as a biomarker in different diseases [25] such as arterial hypertension, which has effects on the secretory capacity of the CP, resulting in lower transferrin levels in the CSF of the SHR with respect to WKY.

The *α*-2-Heremans-Schmid glycoprotein (*α*-2-HS), also known as fetuin-A, is present in the serum being synthesized by hepatocytes, but the *α*-2-HS is also synthesized in the CP and secreted into the CSF, where its principal function is to inhibit pathologic calcifications, since it is capable of forming complexes with calcium and phosphorus [26]. The *α*-2-HS are lower in the CSF of the SHR (Table 1); this decrease could be due to damage in the CP secretions. By contrast, the vitamin D binding protein is higher in the CSF of the SHR. The association between the vitamin D binding protein and hypertension is not clear, but it is thought that this protein, because it is linked to the inflammatory process, could be higher since high blood pressure produces an increase of the inflammatory process as a consequence of the brain damage [27, 28]. Three isoforms of *α*-1-antitrypsin are also higher in the CSF of the SHR when compared to the CSF of the control; such an increase could be due to the three isoforms being implicated in the inflammatory processes which are present in degenerative diseases and in SHR [28–31]. Kininogens are other proteins that are also implicated in inflammation [32], and in the present study kininogens were found to be lower in the CSF of the SHR than in the CSF of the control groups. The study here reports many variations of the proteins participating in inflammatory processes present in SHR; therefore, these results agree with the work of other authors [27, 28] who have reported that hypertension in SHR rats produces several glycoprotein alterations. Differences in other proteins such as *α*-1-*β* glycoprotein were found, which have not been directly related to the development of hypertension, but which have been proposed as biomarkers of diseases with inflammatory processes [29, 33].

This paper reports that the effects of high blood pressure in the brain can be observed in the CSF composition, where two different proteomic profiles were found between SHR and WKY. Among the diverse proteins found, the most noteworthy are *α*-1 antitrypsin, apolipoprotein A1, apolipoprotein E, transthyretin, albumin, *α*-2-HS glycoprotein, transferrin, *α*-1-*β* glycoprotein, IgG, kininogen, vitamin D binding protein, and haptoglobin. Therefore, one could conclude that the high blood pressure in SHR produces CSF variations of proteins that are connected with the blood-to-CSF barrier integrity, cholesterol metabolism, and inflammatory processes, which could be a cause or consequence of choroid plexus alterations and the blood-to-CSF barrier disruption.

## Figures and Tables

**Table 1:** List of proteins detected in CSF that are altered, high or low in SHR compared to WKY using 2*D* electrophoresis and identified by mass spectrometry as described in Section 2.

ID	Protein	NCBI nr	Theoretical MW (Da)/pI	Matched peptide	Sequence coverage (%)	Mascot score	Missed cleavage	Fold SHR expression
1a	*α*-1-Antitrypsin	gi:112889	46.278/5.70	8	22	82	1	**+1.75**
1b	*α*-1-Antitrypsin	gi:112889	46.278/5.70	9	27	81	1	**+2.95**
1c	*α*-1-Antitrypsin	gi:112889	46.278/5.70	9	25	83	1	**+2.60**
2a	Apolipoprotein A1	gi:178775	28.070/5.27	*Identification from Finehout et al., 2004 [12]				**+5.37**
2b	Apolipoprotein A1	gi:178775	28.070/5.27	*Identification from Finehout et al., 2004 [12]				**+5.79**
3	Apolipoprotein E	gi:1703338	35.788/5.23	9	27	78	0	**−1.92**
4a	Transthyretin	gi:136467	15.824/5.77	5	43	97	1	**−2.86**
4b	Transthyretin	gi:136467	15.824/5.77	7	59	85	1	**−3.54**
5	Albumin	gi:55391508	70.682/6.09	13	25	190	1	**+1.41**
6	*α*-2-HS Glycoprotein	gi:231468	38.757/6.05	5	21	85	0	**−1.32**
7	Transferrin	gi:122066515	78.512/7.14	12	22	169	1	**−2.04**
8	*α*-1-*β* Glycoprotein	gi:23503038	51.870/5.51	*Identification from Finehout et al., 2004 [12]				**−3.26**
9	Ig gamma-2A chain C region	gi:121052	35.677/7.72	3	11	59	1	**+1.79**
10	Kininogen	gi:60293582	48.828/6.08	6	16	63	1	**−1.52**
11a	Vitamin D Binding Protein	gi:139643	55.106/5.65	14	34	166	1	**+2.21**
11b	Vitamin D Binding Protein	gi:139643	55.106/5.65	8	21	55	1	**+1.24**
12a	Haptoglobin	gi:59808182	39.052/6.10	8	22	89	1	**+2.67**
12b	Haptoglobin	gi:59808182	39.052/6.10	5	11	63	0	**+3.42**
13	Carbonic anhydrase II	gi:9506445	29.698/6.9	6	33	121	0	**−2.83**
14	Alpha-1-macroglobulin	gi:205384	168.422/6.46	10	8	71	1	**+1.83**
